# Incorporation of Putative Helix-Breaking Amino Acids in the Design of Novel Stapled Peptides: Exploring Biophysical and Cellular Permeability Properties

**DOI:** 10.3390/molecules24122292

**Published:** 2019-06-20

**Authors:** Anthony W. Partridge, Hung Yi Kristal Kaan, Yu-Chi Juang, Ahmad Sadruddin, Shuhui Lim, Christopher J. Brown, Simon Ng, Dawn Thean, Fernando Ferrer, Charles Johannes, Tsz Ying Yuen, Srinivasaraghavan Kannan, Pietro Aronica, Yaw Sing Tan, Mohan R. Pradhan, Chandra S. Verma, Jerome Hochman, Shiying Chen, Hui Wan, Sookhee Ha, Brad Sherborne, David P. Lane, Tomi K. Sawyer

**Affiliations:** 1 MSD International, 8 Biomedical Grove, #04-01/05 Neuros Building, Singapore 138665, Singapore; kristal.kaan@merck.com (H.Y.K.K.); angela.juang@merck.com (Y.-C.J.); ahmad.sadruddin@merck.com (A.S.); shuhui.lim@merck.com (S.L.); 2 p53Lab, Agency for Science, Technology and Research (A*STAR), 8A Biomedical Grove, #06-04/05 Neuros/Immunos, Singapore 138648, Singapore; CJBrown@p53lab.a-star.edu.sg (C.J.B.); simon.ng@merck.com (S.N.); dthean@p53Lab.a-star.edu.sg (D.T.); fjferrer@p53Lab.a-star.edu.sg (F.F.); dplane@p53Lab.a-star.edu.sg (D.P.L.); 3 Institute of Chemical and Engineering Sciences (ICES), Agency for Science, Technology and Research (A*STAR), 8 Biomedical Grove, #07, Neuros Building, Singapore 138665, Singapore; charles_johannes@p53lab.a-star.edu.sg (C.J.); yuenty@ices.a-star.edu.sg (T.Y.Y.); 4 Bioinformatics Institute (BII), Agency for Science, Technology and Research (A*STAR), 30 Biopolis Street, #07-01 Matrix, Singapore 138671, Singapore; raghavk@bii.a-star.edu.sg (S.K.); pietroa@bii.a-star.edu.sg (P.A.); tanys@bii.a-star.edu.sg (Y.S.T.); mohanrp@bii.a-star.edu.sg (M.R.P.); chandra@bii.a-star.edu.sg (C.S.V.); 5 Merck & Co., Inc., West Point, PA 19486, USA; jerome_hochman@merck.com; 6 Merck & Co., Inc., Kenilworth, NJ 07033, USA; shiying.chen@merck.com (S.C.); hui.wan@merck.com (H.W.); sookhee_ha@merck.com (S.H.); tomi.sawyer@merck.com (B.S.); 7 Merck & Co., Inc., Boston, MA 02115, USA

**Keywords:** peptide permeability, stapled peptide, macrocyclic peptide, D-amino acid, helix-breaker

## Abstract

Stapled α-helical peptides represent an emerging superclass of macrocyclic molecules with drug-like properties, including high-affinity target binding, protease resistance, and membrane permeability. As a model system for probing the chemical space available for optimizing these properties, we focused on dual Mdm2/MdmX antagonist stapled peptides related to the p53 N-terminus. Specifically, we first generated a library of ATSP-7041 (Chang et al., 2013) analogs iteratively modified by L-Ala and D-amino acids. Single L-Ala substitutions beyond the Mdm2/(X) binding interfacial residues (i.e., Phe^3^, Trp^7^, and Cba^10^) had minimal effects on target binding, α-helical content, and cellular activity. Similar binding affinities and cellular activities were noted at non-interfacial positions when the template residues were substituted with their d-amino acid counterparts, despite the fact that d-amino acid residues typically ‘break’ right-handed α-helices. d-amino acid substitutions at the interfacial residues Phe^3^ and Cba^10^ resulted in the expected decreases in binding affinity and cellular activity. Surprisingly, substitution at the remaining interfacial position with its d-amino acid equivalent (i.e., Trp^7^ to d-Trp^7^) was fully tolerated, both in terms of its binding affinity and cellular activity. An X-ray structure of the d-Trp^7^-modified peptide was determined and revealed that the indole side chain was able to interact optimally with its Mdm2 binding site by a slight global re-orientation of the stapled peptide. To further investigate the comparative effects of d-amino acid substitutions we used linear analogs of ATSP-7041, where we replaced the stapling amino acids by Aib (i.e., *R*8^4^ to Aib^4^ and *S*5^11^ to Aib^11^) to retain the helix-inducing properties of α-methylation. The resultant analog sequence Ac–Leu–Thr–Phe–Aib–Glu–Tyr–Trp–Gln–Leu–Cba–Aib–Ser–Ala–Ala–NH_2_ exhibited high-affinity target binding (Mdm2 K_d_ = 43 nM) and significant α-helicity in circular dichroism studies. Relative to this linear ATSP-7041 analog, several d-amino acid substitutions at Mdm2(X) non-binding residues (e.g., d-Glu^5^, d-Gln^8^, and d-Leu^9^) demonstrated decreased binding and α-helicity. Importantly, circular dichroism (CD) spectroscopy showed that although helicity was indeed disrupted by d-amino acids in linear versions of our template sequence, stapled molecules tolerated these residues well. Further studies on stapled peptides incorporating N-methylated amino acids, l-Pro, or Gly substitutions showed that despite some positional dependence, these helix-breaking residues were also generally tolerated in terms of secondary structure, binding affinity, and cellular activity. Overall, macrocyclization by hydrocarbon stapling appears to overcome the destabilization of α-helicity by helix breaking residues and, in the specific case of d-Trp^7^-modification, a highly potent ATSP-7041 analog (Mdm2 K_d_ = 30 nM; cellular EC_50_ = 600 nM) was identified. Our findings provide incentive for future studies to expand the chemical diversity of macrocyclic α-helical peptides (e.g., d-amino acid modifications) to explore their biophysical properties and cellular permeability. Indeed, using the library of 50 peptides generated in this study, a good correlation between cellular permeability and lipophilicity was observed.

## 1. Introduction

Intracellular protein–protein interactions (PPIs) represent a plethora of potential drug targets across therapeutic classes. However, the discovery of disease-modifying molecules against PPI targets has proceeded slowly due to the limitations of traditional therapeutic strategies [1]. In particular, the large and flat surfaces of PPIs pose challenges for small molecules (<500 daltons) as potent inhibitors [1]. Monoclonal antibodies represent the other major class of therapeutics and are highly effective at inhibiting PPIs, however, methods for efficient intracellular delivery of such molecules have not yet been realized [2]. Peptides also have the capacity to compete with PPIs but generally have poor proteolytic stability and limited membrane permeability, which greatly hinder their therapeutic application to intracellular targets [3]. However, the macrocyclization of peptides has been shown to address both of these liabilities [4]. Most prominently, hydrocarbon stapling of helical peptides has emerged as a potential therapeutic approach with ALRN-6924—a first-generation molecule, currently undergoing clinical trials [5]. Hydrocarbon stapling generally involves covalently linking two α-methyl, olefin-containing amino acids on the same helical face (typically either at i to i +4 or i to i +7 positions) via ring closing metathesis [6,7,8]. Such hydrocarbon stapling (or other macrocyclization chemistries) has been reported to confer drug-like properties, including stabilization of the peptide into an α-helical conformation, increased target binding affinity, enhanced proteolytic stability and/or improved membrane permeability [4].

**Figure 1:**
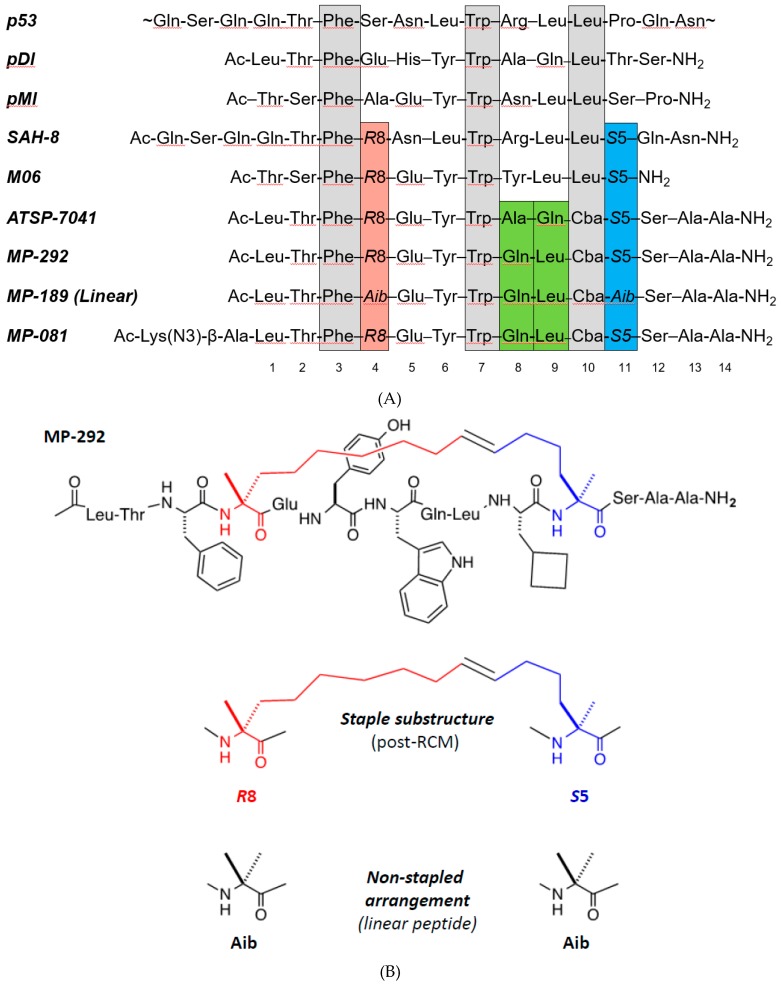
Template sequences used in this study and comparison to related literature peptides. (**A**) Sequences of p53; phage display peptides pDI and pMI; literature stapled peptides SAH-8, M06, and ATSP-7041; and ATSP-7041 variants used as template sequences in this work, MP-292, MP-189, and MP-018. (**B**) Chemical structure of MP-292, as well as the substructure of the stapling amino acids (*R*8 and *S*5) and Aib.

Although there have been several examples of stapled peptides with activity in cellular systems, most peptides reported thus far have marginal cellular permeability, as evidenced by the large disparity between their target binding biochemical and target-dependent cellular functional potencies. Therefore, a deeper understanding of the design rules governing stapled peptide cellular permeability would greatly enable their development as potential clinical candidates. Exploration of the peptide permeability determinants may be facilitated by expanded chemical diversity. Here, we show that α-helical stapled peptides unexpectedly tolerate residues that are generally considered α-helix breakers within the context of linear sequences. To study this, we used ATSP-7041 analogs [9]—stapled peptides with sequences related to the p53 N-terminus [10], those discovered by phage display (pDI [11] and pMI [12]), and with similarity to the literature staple peptides SAH-8 [13] and M06 [14]. Stapled peptide analogs harboring helix-breakers, such as d-amino acids, Pro, Gly, and N-methylated amino acids, generally retained their α-helical structure, binding affinity for Mdm2, and cellular functional activity (See Figure 1A,B for the sequence and structure of peptide templates used in this study). Overall, our work shows for the first time a compelling structural and chirality tolerance of macrocyclic α-helical peptides and provides impetus for future exploration of their biophysical and cellular permeability properties.

## 2. Methods

### 2.1. Peptide Synthesis

Peptides were synthesized using Rink Amide MBHA resin and Fmoc-protected amino acids, coupled sequentially with DIC/HOBt activating agents. Double coupling reactions were performed on the first amino acid and also at the stapling positions. At these latter positions, the activating reagents were switched to DIEA/HATU for better coupling efficiencies. Ring closing metathesis reactions were performed by first washing the resin three times with DCM, followed by the addition of the first-generation Grubbs Catalyst (20 mol % in DCM and allowed to react for 2 h; all steps with Grubbs Catalyst were performed in the dark). The RCM (ring closing metathesis) reaction was repeated to ensure a complete reaction. After the RCM was complete, a test cleavage was performed to ensure adequate yield. Peptides were cleaved and then purified as a mixture of cis-trans isomers by RP-HPLC.

### 2.2. Protein Production

For use in the peptide binding assay, a human Mdm2 1–125 sequence was cloned into a pNIC-GST vector. The TEV (tobacco etch virus) cleavage site was changed from ENLYFQS to ENLYFQG to give a fusion protein with the following sequence:

MSDKIIHSPILGYWKIKGLVQPTRLLLEYLEEKYEEHLYERDEGDKWRNKKFELGLEFPNLPYYIDGDVKLTQSMAIIRYIADKHNMLGGCPKERAEISMLEGAVLDIRYGVSRIAYSKDFETLKVDFLSKLPEMLKMFEDRLCHKTYLNGDHVTHPDFMLYDALDVVLYMDPMCLDAFPKLVCFKKRIEAIPQIDKYLKSSKYIAWPLQGWQATFGGGDHPPKLEVLFQGHMHHHHHHSSGVDLGTENLYFQGMCNTNMSVPTDGAVTTSQIPASEQETLVRPKPLLLKLLKSVGAQKDTYTMKEVLFYLGQYIMTKRLYDEKQQHIVYCSNDLLGDLFGVPSFSVKEHRKIYTMIYRNLVVVNQQESSDSGTSVSEN-.

The corresponding plasmid was transformed into BL21 (DE3) Rosetta T1R *Escherichia coli* cells and grown under kanamycin selection. Bottles of 750 mL Terrific Broth, supplemented with appropriate antibiotics and 100 μL of antifoam 204 (Sigma-Aldrich, St. Louis, MO, USA, were inoculated with 20 mL seed cultures grown overnight. The cultures were incubated at 37 °C in the LEX system (Harbinger Biotech, Toronto, Canada) with aeration and agitation through the bubbling of filtered air through the cultures. The LEX system temperature was reduced to 18 °C when culture OD600 reached 2, and the cultures were induced after 60 min with 0.5 mM IPTG. Protein expression was allowed to continue overnight. Cells were harvested by centrifugation at 4000× *g*, at 15 °C for 10 min. The supernatants were discarded and the cell pellets were resuspended in a lysis buffer (1.5 mL per gram of cell pellet). The cell suspensions were stored at −80 °C before purification work.

The re-suspended cell pellet suspensions were thawed and sonicated (Sonics Vibra-Cell, Newtown, CO, USA) at 70% amplitude, 3 s on/off for 3 min, on ice. The lysate was clarified by centrifugation at 47,000× *g*, 4 °C for 25 min. The supernatants were filtered through 1.2 μm syringe filters and loaded onto the AKTA Xpress system (GE Healthcare, Fairfield, CO, USA). The purification regime is briefly described as follows. The lysates were loaded onto a 1 mL Ni-NTA Superflow column (Qiagen, Valencia, CA, USA) that had been equilibrated with 10 column volumes of wash 1 buffer. Overall buffer conditions were as follows: Immobilized metal affinity chromatography (IMAC) wash 1 buffer—20 mM HEPES ((4-(2-hydroxyethyl)-1-piperazineethanesulfonic acid), 500 mM NaCl, 10 mM Imidazole, 10% (*v*/*v*) glycerol, 0.5 mM TCEP (Tris(2-carboxyethyl)phosphine), pH 7.5; IMAC wash 2 buffer—20 mM HEPES, 500 mM NaCl, 25 mM Imidazole, 10% (*v*/*v*) glycerol, 0.5 mM TCEP, pH 7.5; IMAC Elution buffer—20 mM HEPES, 500 mM NaCl, 500 mM Imidazole, 10% (*v*/*v*) glycerol, 0.5 mM TCEP, pH 7.5. The sample was loaded until air was detected by the air sensor, 0.8 mL/min. The column was then washed with wash 1 buffer for 20 column volumes, followed by 20 column volumes of wash 2 buffer. The protein was eluted with five column volumes of elution buffer. The eluted proteins were collected and stored in sample loops on the system and then injected into gel filtration (GF) columns. Elution peaks were collected in 2 mL fractions and analyzed on SDS-PAGE gels. The entire purification was performed at 4 °C. Relevant peaks were pooled, TCEP was added to a total concentration of 2 mM. The protein sample was concentrated in Vivaspin 20 filter concentrators (VivaScience, Littleton, MA, USA) at 15 °C to approximately 15 mg/mL. (<18 kDa—5 K MWCO, 19–49 kDa—10 K MWCO, >50 kDa—30 K MWCO). The final protein concentration was assessed by measuring absorbance at 280 nm on Nanodrop ND-1000 (Thermo Fisher, Waltham, MA, USA). The final protein purity was assessed on SDS-PAGE gel. The final protein batch was then aliquoted into smaller fractions, frozen in liquid nitrogen and stored at −80 °C.

For x-ray crystallography, Mdm2 (6–125) was cloned as a GST-fusion protein using the pGEX-6P-1 GST expression vector (GE Healthcare). The GST-fused Mdm2 (6–125) construct was then transformed into *Escherichia coli* BL21(DE3) pLysS (Thermo Fisher, Waltham, MA, USA) competent cells. Cells were grown in Luria-Bertani (LB) medium at 37 °C and induced at OD600 nm of 0.6 with 0.5 mM Isopropyl β- D -1-thiogalactopyranoside (IPTG) at 16 °C. After overnight induction, the cells were harvested by centrifugation, resuspended in binding buffer (50 mM Tris-HCl pH 8.0, 150 mM NaCl), and lysed by sonication. After centrifugation for 60 min at 19,000× *g* at 4 °C, the cell lysate was then applied to a 5 mL GSTrap FF column (GE Healthcare) pre-equilibrated in wash buffer (50 mM Tris-HCl pH 8.0, 150 mM NaCl, 1 mM DTT). The GST-fused Mdm2 (6–125) was then cleaved on-column by PreScission protease (GE Healthcare) overnight at 4 °C and eluted off the column with wash buffer. The protein sample was then dialyzed into a buffer A solution (20 mM Bis-Tris, pH 6.5, 1 mM DTT) using HiPrep 26/10 Desalting column, and loaded onto a cation-exchange Resource S 1 mL column (GE Healthcare), pre-equilibrated in buffer A. The column was then washed in six column volumes of buffer A and the bound protein was eluted with a linear gradient in buffer comprising 1 M NaCl, 20 mM Bis-Tris pH 6.5, and 1 mM DTT over 30 column volumes. Protein purity as assessed by SDS-PAGE was ~95%, and the proteins were concentrated using Amicon-Ultra (3 kDa MWCO) concentrator (Millipore, Burlington, MA, USA). Protein concentration was determined using 280 nm absorbance measurements.

### 2.3. Crystallization and Data Collection

Mdm2 (6-125) was concentrated to 3.5 mg/mL and then incubated with the stapled peptide at a 1:3 molar ratio of protein to peptide at 4 °C overnight. The lyophilized stapled peptide (MP-594) was first dissolved in dimethyl sulfoxide (DMSO) to make a 100 mM stock solution before being directly added to the protein solutions. The sample was clarified by centrifugation before crystallization trials at 16 °C using the sitting drop vapor diffusion method. Crystals of Mdm2 (6–125) in complex with MP-594 were obtained by mixing the protein–peptide complex with the reservoir solution in a ratio of 1:1, with the reservoir solution containing 2.4 M di-sodium malonate. Mdm2/MP-594 complex crystals were frozen in an equivalent mother liquor solution containing 15% (*v*/*v*) glycerol and then flash frozen in liquid nitrogen. X-ray diffraction was collected at the Australian synchrotron (Aus.). See Table S1 for data collection statistics.

### 2.4. X-ray Structure and Refinement

X-ray datasets were processed and scaled with the XDS1 and CCP42 packages [15,16]. The structures were solved by molecular replacement with the program PHASER3, using the human Mdm2 (6-125) structure from the PDB:4UMN (chain A) as a search model [17]. The starting model was built and refined by iterative cycles of manual and automatic building with Coot4 and restrained refinement with Refmac5 [18,19]. The geometric restraints for the non-natural amino acids constituting the hydrocarbon staple and the covalent bond linking their respective side chains together, to form the macrocyclic linkage constraining the stapled peptide, were defined and generated using JLigand6 [20]. The final model was validated using RAMPAGE7 [21] and the MOLPROBITY8 [21] webserver. Structural overlays and analysis was performed using PYMOL software (Schrödinger, New York, NY, USA). See Table S1 for data collection and refinement statistics. The Mdm2: MP-594 complex structure was deposited in the PDB with the accession code 6AAW.

### 2.5. Mdm2 Binding Assay

Purified Mdm2 (1–125) protein was titrated against 50 nM carboxyfluorescein (FAM)—labeled 12/1 peptide13 (FAM-RFMDYWEGL-NH2). Dissociation constants for titration of Mdm2 against the FAM-labeled 12/1 peptide were determined by fitting the experimental data to a 1:1 binding model equation, shown below:

(1) $$r=r_{0}+\left(r_{b}-r_{0}\right)\times \frac{\left(K_{d}+[L{]}_{t}+[P{]}_{t}\right)-\sqrt{{\left(K_{d}+[L{]}_{t}+[P\right]}_{t}{)}^{2}-4[L{]}_{t}[P{]}_{t}}}{2{\left[L\right]}_{t}}$$

where [*P*] is the protein concentration (Mdm2), [*L*] is the labeled peptide concentration, *r* is the anisotropy measured, *r_0_* is the anisotropy of the free peptide, *r_b_* is the anisotropy of the Mdm2–FAM-labeled peptide complex, *K_d_* is the dissociation constant, [*L*]*_t_* is the total FAM-labeled peptide concentration, and [*P*]*_t_* is the total Mdm2 concentration. The determined apparent *K_d_* value of FAM-labeled 12/1 peptide (13.0 nM) was used to determine the apparent *K_d_* values of the respective competing ligands in subsequent competition assays in fluorescence anisotropy experiments. Titrations were carried out with the concentration of Mdm2 held constant at 250 nM and the labeled peptide at 50 nM. The competing molecules were then titrated against the complex of the FAM-labeled peptide and protein. Apparent *K_d_* values were determined by fitting the experimental data to the equations shown below:

(2) $$r=r_{0}+\left(r_{b}-r_{0}\right)\times \frac{2\sqrt{\left(d^{2}-3e\right)\cos\left(\theta /3\right)-9}}{3K_{d1}+2\sqrt{(d^{2}}-3e)\cos\left(\theta /3\right)-d}\;d=K_{d1}+K_{d2}+[L{]}_{st}+[L{]}_{t}-{\left[P\right]}_{t}\;e=({\left[L\right]}_{t}-\left[P{]}_{t}\right)K_{d1}+({\left[L\right]}_{st}-\left[P{]}_{t}\right)K_{d2}+K_{d1}K_{d2}\;f=-K_{d1}K_{d2}{\left[P\right]}_{t}\;\theta =ar\cos[\frac{-2d^{3}+9de-27f}{2\sqrt{{\left(d^{2}-3e\right)}^{3}}}]$$

where [*L*]*_st_* and [*L*]*_t_* denote labeled ligand and total unlabeled ligand input concentrations, respectively. *K_d2_* is the dissociation constant of the interaction between the unlabeled ligand and the protein. In all competition experiments, it was assumed that [*P*]*_t_* > [*L*]*_st_*, otherwise considerable amounts of free labeled ligand would always be present and would interfere with measurements. *K_d1_* is the apparent *K_d_* for the labeled peptide used and was experimentally determined as described in the previous paragraph. The FAM-labeled peptide was dissolved in dimethyl sulfoxide (DMSO) at 1 mM and diluted into the experimental buffer. Readings were carried out with an Envision Multilabel Reader (PerkinElmer). Experiments were carried out in PBS (2.7 mM KCl, 137mM NaCl, 10 mM Na2HPO4, and 2 mM KH2PO4 (pH 7.4)) and 0.1% Tween 20 buffer. All titrations were carried out in triplicate. Curve-fitting was carried out using Prism 4.0 (GraphPad). To validate the fitting of a 1:1 binding model, we carefully ensured that the anisotropy value at the beginning of the direct titrations between Mdm2 and the FAM-labeled peptide did not differ significantly from the anisotropy value observed for the free fluorescently-labeled peptide. Negative control titrations of the ligands under investigation were also carried out with the fluorescently labeled peptide (in the absence of Mdm2) to ensure no interactions were occurring between the ligands and the FAM-labeled peptide. In addition, we ensured that the final baseline in the competitive titrations did not fall below the anisotropy value for the free FAM-labeled peptide, which would otherwise indicate an unintended interaction between the ligand and the FAM-labeled peptide to be displaced from the Mdm2 binding site.

### 2.6. p53 Beta-Lactamase Reporter Gene Cellular Functional Assay

HCT116 cells were stably transfected with a p53 responsive β-lactamase reporter and were seeded into a 384-well plate at a density of 8000 cells per well. Cells were maintained in McCoy’s 5A Medium with 10% fetal bovine serum (FBS), Blasticidin, and Penicillin/Streptomycin. The cells were incubated overnight, followed by removal of cell growth media and replacement with Opti-MEM either containing 0% FBS or 10% FBS. Peptides were then dispensed to each well using a liquid handler, ECHO 555, and incubated for 4 or 16 h. The final working concentration of DMSO was 0.5%. β-lactamase activity was detected using the ToxBLAzer Dual Screen (Invitrogen), as per the manufacturer’s instructions. Measurements were made using the Envision multiplate reader (Perkin–Elmer). Maximum p53 activity was defined as the amount of β-lactamase activity induced by 50 µM azide-ATSP-7041. This was determined as the highest amount of p53 activity induced by azide-ATSP-7041 from titrations on HCT116 cells.

### 2.7. Lactate Dehydrogenase (LDH) Release Assay

HCT116 cells were seeded into a 384-well plate at a density of 8000 cells per well. Cells were maintained in McCoy’s 5A Medium with 10% fetal bovine serum (FBS), Blasticidin, and Penicillin/Streptomycin. The cells were incubated overnight, followed by the removal of cell media and the addition of Opti-MEM Medium without FBS. Cells were then treated with peptides for 4 or 16 h in Opti-MEM, either in 10% FBS or in serum free conditions. The final concentration of DMSO was 0.5%. Lactate dehydrogenase release was detected using the CytoTox-ONE Homogenous Membrane Integrity Assay Kit (Promega), as per the manufacturer’s instructions. Measurements were carried out using the Tecan plate reader. Maximum LDH release was defined as the amount of LDH released as induced by the lytic peptide (iDNA79) and used to normalize the results.

### 2.8. Tetracycline Beta-Lactamase Reporter Gene Cellular Assay (Counterscreen)

This assay was based on Jump-In™ T-REx™ CHO-K1 BLA cells containing a stably integrated β-lactamase under the control of an inducible cytomegalovirus (CMV) promoter. Cells were seeded into a 384-well plate at a density of 4000 cells per well. Cells were maintained in Dulbecco’s Minimal Eagle Medium (DMEM) with 10% fetal bovine serum (FBS), Blasticidin, and Penicillin/Streptomycin. The cells were incubated for 24 h, followed by cell media removal and replacement with Opti-MEM, either containing 10% FBS or 0% FBS. Peptides were then dispensed to each well using a liquid handler, ECHO 555 and incubated for 4 or 16 h. The final working concentration of DMSO was 0.5%. β-lactamase activity was detected using the ToxBLAzer Dual Screen (Invitrogen), as per the manufacturer’s instructions. Measurements were carried out using the Envision multiplate reader (Perkin–Elmer). Counterscreen activity was defined as the amount of β-lactamase activity induced by tetracycline.

### 2.9. Isothermal Titration Calorimetry (ITC)

Overnight dialysis of protein and peptides were carried out in buffer containing 1× phosphate-buffered saline (PBS) pH 7.2, 3% DMSO, and 0.001% Tween-20. Approximately 100–200 µM of peptide was titrated into 20 µM of purified recombinant human Mdm2 protein (amino acids 1–125), over 40 injections of 1 µL each. Reverse ITC (200 µM of Mdm2 protein titrated into 20 µM of peptide) was carried out for peptides that are insoluble at high concentrations. All experiments were performed in duplicates using the MicroCal PEAQ-ITC Automated system. Data analysis was carried out using the MicroCal PEAQ-ITC Analysis Software.

### 2.10. Circular Dichroism (CD)

A total of 5 µL of the 10 mM stock peptide was mixed with 45 µL of 100% methanol, and dried for 2 h in the SpeedVac concentrator (Thermo Scientific). The dried peptide was reconstituted in a buffer (1 mM Hepes pH 7.4 and 5% methanol) to a concentration of 1 mM. The peptide sample was placed in a quartz cuvette with a path length of 0.2 cm. The peptide concentration was determined by the absorbance of the peptide at 280 nM. The CD spectrum was recorded from 300 to 190 nm using the Chirascan-plus qCD machine (Applied Photophysics, Surrey, UK), at 25 °C. All experiments were done in duplicates. The CD spectrum was converted to mean residue ellipticity, before deconvolution and estimation of the secondary structure components of the peptide using the CDNN software (distributed by Applied Photophysics).

### 2.11. Surface Plasmon Resonance (SPR)

SPR experiments were performed with Biacore T100 (GE Healthcare) at 25 °C. The site-specific mono-biotinylated Mdm2 was prepared by sortase-mediated ligation. The SPR buffer consisted of 50 mM Tris pH 7.4, 150 mM NaCl, 1 mM DTT, 0.05% Tween 20, and 3% DMSO. The CM5 chip was first conditioned with 100 mM HCl, followed by 0.1% SDS, 50 mM NaOH, and then water, all performed twice with 6 sec injection at a flow rate of 100 µL/min. With the flow rate set to 10 µL/min, streptavidin (S4762, Sigma-Aldrich) was immobilized on the conditioned chip through amine coupling, as described in the Biacore manual. Excess protein was removed by 30 s injection of the wash solution (50 mM NaOH + 1 M NaCl) at least eight times. The immobilized level was ~3000 RU. The biotinylated Mdm2 was captured by streptavidin, up to a level of ~400 RU. A flow cell consisting of only streptavidin was used as the reference surface. Using a flow rate of 30 µL/min, peptides dissolved in the SPR buffer were injected for 180 s. The dissociation was monitored for 300 s. For each peptide concentration, the peptide injection was followed by a similar injection of SPR buffer to allow the surface to be fully regenerated (though not completely for peptides with an extremely slow off-rate). After the run, responses from the target protein surface were transformed by: (i) correcting with the DMSO calibration curve, (ii) subtracting the responses obtained from the reference surface, and (iii) subtracting the responses of the buffer injections from those of peptide injections. The last step is known as double referencing, which corrects the systematic artefacts. The resulting responses were subjected to kinetic analysis by global fitting with a 1:1 binding model to obtain the *KD*, *k_a_* (M-1 s-1), and *k_d_* (s-1). Binding responses that did not have enough curvature during the association and dissociation were subjected to steady-state analysis, and the *KD* was obtained by fitting a plot of response at equilibrium against the concentration.

### 2.12. Computational Chemistry and Molecular Dynamics (MD) Studies

The crystal structure of the stapled peptide ATSP-7041 co-crystalized with the N-terminal domain of MdmX (PDB ID 4N5T) [9] was used to generate a model of ATSP-7041 bound to the N-terminal domain of Mdm2; the sequence identity between Mdm2 and MdmX was ~53% in their N-terminal domains. Following the mutation of Ala^8^-Gln^9^ to Gln^8^-Leu^9^, the stapled peptide analog (MP-292) and its complex with Mdm2 were used for generating all the systems explored in this study and for the MD simulations. The Xleap module of the program AMBER16 [22] was used to prepare the systems and the N- and C- termini of the peptide and Mdm2 were acetylated and amidated respectively. The parameters for the staple linkers were taken from our previous study [23]. All the simulation systems were prepared and simulated using protocols, as previously described [23]. MD simulations were carried out in triplicate for 250 ns each, using the pemed.cuda module from the AMBER 16 package in combination with the ff14SB force field [24]. To enhance the conformational sampling, each of these peptides was subjected to biasing potential replica exchange MD (BP-REMD) simulations [25] using eight replicas for 50 ns. Simulation trajectories were visualized using the program VMD [26] and images were generated using the program Pymol [27].

### 2.13. Whole Cell Homogenate Stability

Peptides at a concentration of 1 μM were incubated at 37 °C with HCT116 whole cell homogenates prepared from 1 million lysed cells/mL. The reaction was stopped at 0, 1, 2, 4, and 22 h with an organic solvent followed by centrifugation. The resulting supernatant was injected into LC/MS for detection of the tested peptide. The remaining percentage of each compound was normalized to the 0 h amount and reported. As a positive control, we used the ONEG peptide with the sequence PLGRPQLRRGQF [28].

### 2.14. Stapled Peptide Lipophilicity Analysis and Correlation with Cellular/Target Ratios

To explore the correlation between the stapled peptide lipophilicity and cellular permeability, two methods were adapted from the literature: HPLC-LogD [29] and ALogP [30,31]. As related to macrocyclic peptides, such experimental and predictive lipophilicity may be understood in terms of cellular uptake mechanisms ranging from passive permeability [32] to active transport (e.g., endocytosis and/or translocation) [33,34]. The correlation of HPLC-LogD and ALogP data to cellular potency normalized to target binding affinity (cellular EC_50_/Mdm2 K_d_) was then determined to explore whether lipophilicity may be a contributing factor to cellular permeability for the stapled peptides tested here.

### 2.15. Statistics

Experimental data was collected in at least triplicate and coefficients of variations (% CV) were all <30%. Binding and cellular potency values are reported as geometric means, whereas circular dichroism spectroscopy values are reported as arithmetic means.

## 3. Results and Discussion

### 3.1. MP-292 is a High Affinity Mdm2 Binder with On-Target Cellular Activity

**Figure 2:**
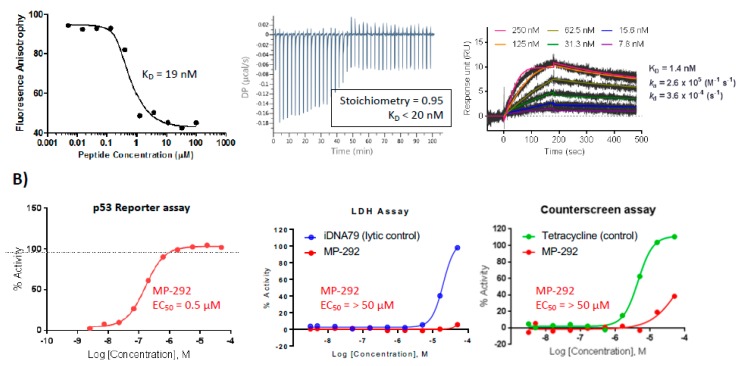
MP-292 is a bona fide Mdm2 binder with on-target cellular activity. (**A**) MP-292 binds to recombinant Mdm2 in a host of biophysical assays, including competitive fluorescence polarization (left panel), isothermal titration calorimetry (middle panel), and surface plasmon resonance (right panel). (**B**) MP-292 shows cellular activity in a β-lactamase-based p53 reporter assay (left panel) but not in counterscreen assays, such as those probing for disruption in membrane integrity (lactate dehydrogenase release, middle panel), nor a β-lactamase based reporter-assay driven from a tetracycline-dependent promoter (p53 independent, right panel).

To explore the effects of various residue types on stapled peptide secondary structure, binding affinity, and cellular activity, we selected MP-292, an analog of ATSP-7041 but with a substitution of the Ala^8^-Gln^9^ residues with Gln^8^-Leu^9^ (Figure 1A). To ensure stoichiometric binding, as well as on-target cellular activity [35], we applied a combination of competitive fluorescence polarization (FP), surface plasmon resonance (SPR), and isothermal titration calorimetry (ITC). Specific and high-affinity binding of MP-292 for Mdm2 was demonstrated through the ability of unlabeled peptides to compete for the fluorescent tracer by FP, canonical saturable SPR sensorgrams [36,37], and 1:1 stoichiometric binding by ITC (Figure 2A). In our cell-based p53 reporter assay, we determined this peptide to have a sub-micromolar EC_50_ in 0% serum (0.5 µM, Figure 2B) and with slightly weaker potencies in 2% and 10% serum (0.7 µM and 4.2 µM, respectively, data not shown). Critically, on-target cellular activity of this molecule was demonstrated, as MP-292 was inactive in our cellular counterscreen and was not able to compromise the plasma membrane, as it was inactive in our LDH release assay (Figure 2B). Indeed, all the stapled p53 peptides investigated here had EC_50_ values >50 µM in both the counterscreen and LDH assays (data not shown).

### 3.2. Ala Scanning of Stapled Peptide Analogs of MP-292 Confirms Key Residues for Target Binding

**Figure 3:**
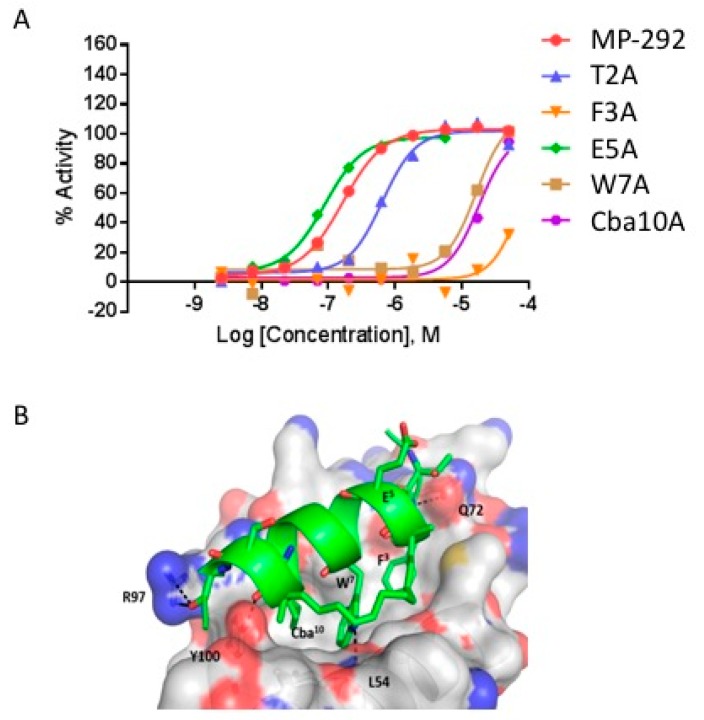
(**A**) Select dose response curves of MP-292 analogs containing Ala substations in the p53 reporter assay. (**B**) Model of MP-292 in complex with Mdm2. Mdm2 is shown as surface (Grey color) and the bound peptide (Green color) is shown as a cartoon. Peptide residues, staple linker, and interacting residues from Mdm2 are shown as lines, with Mdm2/MP-292 hydrogen bond interactions shown as dashed lines (black).

As a first step to explore the tolerance of MP-292 to structural substitutions, we performed Ala scanning substitution across the sequence, except for the *R*8^4^ and *S*5^11^ residues that were critical to the macrocyclization. In agreement with previously reported studies [5,9,10,11], Ala substitution at Phe^3^, Trp^7^, and Cba^10^ decreased Mdm2 binding affinity by approximately 10- to 100-fold, as measured by competitive fluorescence polarization (Table 1A) and surface plasmon resonance (Table S2). Lowered Mdm2 binding affinity was reflected by corresponding losses in cellular potency (Table 1A and Figure 3A). In contrast, Ala substitutions at other residues of MP-292 were well-tolerated, with minimal changes to Mdm2 binding affinities (Table 1A). These results were expected based on prior biophysical studies [5,9,10,11] and an X-ray co-crystal structure of the progenitor peptide ATSP-7041 bound to the MdmX protein [9]. Computational modeling of MP-292 with Mdm2 was consistent with Phe3, Trp7, and Cba10 as the most critical residues for molecular interactions with the target (Figure 3B, see Supplementary Section S1 for a discussion of homology model details). For the substitutions at non-interfacial residues, CD spectroscopy rationalized the lack of affinity changes, since the Ala substitutions showed minimal effects on α-helicity in free solution (Table 1A). Specifically, we determined an average value of approximately 40% α-helicity (ranging from 27.6 to 53.3%) for this stapled peptide series. These α-helicity values were consistent between runs and with distinct methods of sample preparation and concentration determination (data not shown). Computational modeling and MD simulations of the unbound peptides in solution also predicted a moderate level of helicity without any dramatic fluctuations in secondary structures (Figure S1). Further simulations predicted increased α-helicity (>85%) of all the stapled peptides in their Mdm2 bound state (Figure S1).

### 3.3. α-Helicity, Target Binding, and Cellular Activity Were Generally Unaffected by d-Amino Acid Substitutions into MP-292

Considering the helix-inducing propensity of alanine, it was not surprising that Ala substitution at non-interfacial positions of MP-292 had relatively minimal effects on binding affinities and cellular activities. To explore whether more drastic substitutions may be tolerated, we performed a d-amino acid scan of MP-292 (except for the *R*8^4^ and *S*5^11^ residues). As predicted, both d-Phe^3^ and d-Cba^10^ substitutions resulted in stapled peptide analogs exhibiting significantly decreased Mdm2 binding affinities (Table 1B), details of the specific mechanisms for affinity loss were studied by MD simulations and are detailed in the Supplementary Information, Section S2. Unexpectedly, substitution of Trp^7^ by d-Trp^7^ gave an Mdm2 binding affinity and cellular activity similar to that of MP-292 (Table 1B); a result that was explained by biophysical (CD), computational modeling, and X-ray crystallography studies (vide infra). For the non-interfacial residues, d-amino acid substitutions were well-tolerated with respect to Mdm2 binding affinities or cellular activities (Table 1B). A comparative analysis of the α-helical content of the d-amino acid scan peptides with the aforementioned Ala-scan peptides by CD spectroscopy corroborated such biochemical and cellular data. Specifically, it was determined that the α-helical properties of the d-amino acid scan series of MP-292 analogs were similar to those of the Ala-scan peptides (Table 1 and Figures S1 and S2).

### 3.4. d-Amino Acid Scanning of Linear Peptides Results in Decreased α-Helicity, Target Binding, and Cellular Activity

**Figure 4:**
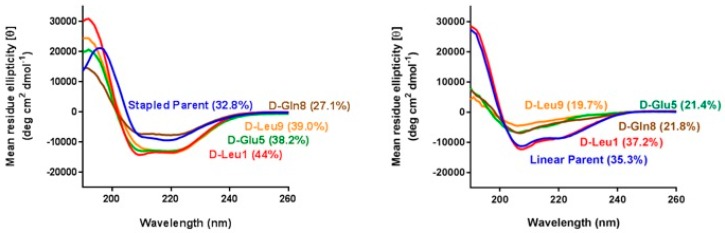
Circular dichroism spectra of stapled peptides bearing d-amino acid substitutions (left panel) and their corresponding linear counterparts (right panel). The table gives the sequences of the parent peptides.

To ascertain if the retention of α-helicity for the d-amino acid peptides was a consequence of conformational stabilization by the *R*8^4^-to-*S*5^11^ hydrocarbon stapling, we designed a series of linear peptides containing Aib^4^ and Aib^11^ substitutions to impart α-helical inducing properties, albeit without the benefit of macrocyclization. With the exception of the d-Leu^1^ substituted analog, the linear peptides containing d-amino acid replacements (e.g., d-Glu^5^, d-Gln^8^, and d-Leu^9^) showed a dramatic loss in α-helical properties, as evidenced by less intense spectra and decrease of the minima at 222 nm (Figure 4), a hallmark of the α-helical conformation. Remarkably, despite the low helical content of the d-amino acid modified linear peptides, as determined by CD analysis, they exhibited relatively potent Mdm2 binding affinities (Table 1C), in line with a previous report, where binding was retained in a linear Mdm2-binding peptide (pMI) with d-amino acid substitutions at the C-terminus [38]. Importantly, and in contrast to the stapled peptide series, the linear peptide analogs possessed marginal, if any, cellular activity (Table 1C), a phenomenon likely due to a combination of compromised intracellular stability (vide infra) and cell permeability.

### 3.5. d-Trp^7^ Modified Stapled Peptide: Computational Modeling and X-Ray Crystallographic Studies with Mdm2

**Figure 5:**
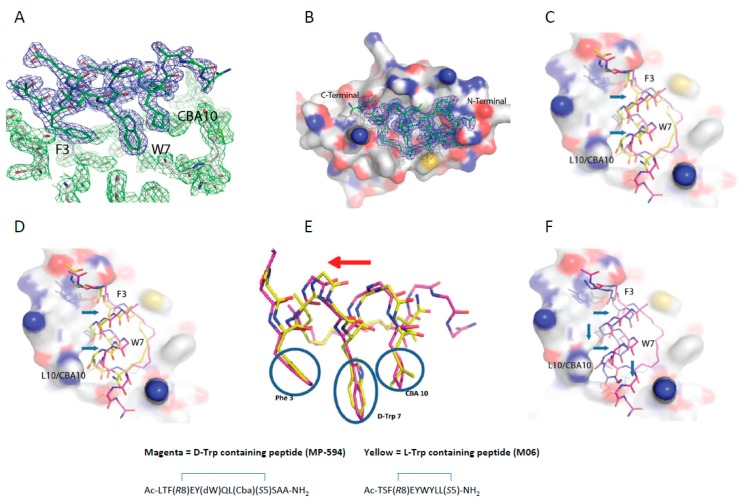
(**A**) The three critical residues (F3, D-Trp^7^, and Cba^10^) responsible for the interaction of the peptide with Mdm2 project into a hydrophobic groove on the surface of Mdm2. 2Fo-Fc map for ATSP-7041 variant with a W^7^ to d-Trp substitution (MP-594) peptide is shown in blue and the 2Fo-Fc map for Mdm2 is shown in green (1.5σ). (**B**) Surface representation of Human Mdm2 (6–125) in complex with MP-594. The 2Fo-Fc electron density map for MP-594 is shown in blue mesh (1.5σ). (**C**) Alignment of MP-594 (magenta) with the M06 stapled peptide (chain C from PDB ID: 4UMN, yellow) (**D**) and the SAH-8 stapled peptide (chain B from PDB ID: 3V3B, cyan), both respectively in complex with Mdm2, highlighting the translation (blue arrows) of MP-594 across the peptide binding groove. (**E**) Overlay of MP-594 and M06, demonstrating that the d-Trp^7^ sidechain of MP-594 occupies the same volume of space as the Trp^7^ in M06 without perturbing the spatial positions of the other critical side chains in relation to each other or the overall α-helical fold of the constrained peptide. Only a global shift in register of the whole peptide occurs to maintain these critical interactions in their optimal positions with Mdm2. (**F**) Alignment of MP-594 (magenta) with the linear pMI N8A peptide (chain B from PDB ID: 3LNZ, blue), both in complex with Mdm2, where gross differences can be observed between both molecules in terms of displacement along and across the Mdm2 binding pocket (blue arrows).

We next sought to understand why d-Trp^7^ substitution at a critical interfacial position for Mdm2 molecular recognition did not abrogate binding. MD simulations showed that the d-Trp^7^ side chain undergoes a re-orientation to adopt a conformation that is highly similar to that adopted by the l-Trp^7^ sidechain in MP-292 (Figure S3, Movies 1,2), and allowed for the retention of critical hydrogen bonds. Given that the d-Trp^7^ sidechain projects off the helical backbone with a significantly different spatial vector, compensating changes were requisite to enable the peptide analog to bind Mdm2 in a productive manner. Specifically, a combination of changes to the Phe^3^ and Cba^10^ sidechain torsion angles and a cooperative shift in α-helical register was predicted to occur to allow the Phe^3^, d-Trp^7^, and Cba^10^ sidechains to bind effectively. This would also preserve a hydrogen bond between the d-Trp^7^ indole nitrogen and the backbone carbonyl oxygen of Leu54. The rationale provided by such computational simulations was confirmed by solving the co-crystal structure of the d-Trp^7^-modified stapled peptide MP-594 in complex with Mdm2 (6–125). The structure revealed a single complex in the asymmetric unit of the crystal. The p53 peptide binding groove in the Mdm2 complex was occupied by a single molecule of MP-594, wherein electron density in the 2Fo-Fc was observed for the entirety of the stapled peptide, interacting with the target protein by burying the residues Phe^3^, d-Trp^7^, and Cba^10^ into the same hydrophobic cleft where Nutlin and p53-derived peptides are known to interact (Figure 5A,B). The d-Trp^7^-substituted stapled peptide aligns itself along the p53 peptide binding site in the same orientation as other stapled peptides co-crystallized with Mdm2, including M06 (PDB: 4UMN) and SAH-8 (PDB: 3V3B) (Figure 5C,D). Interestingly, when the Mdm2:MP-594 complex is overlaid with the Mdm2:M06 complex (Figure 5E), the d-Trp^7^ sidechain is observed to occupy the same volume of space as the Trp in M06 without perturbing the spatial positions of the other critical side chains in relation to each other (Figure 5E) or the overall α-helical fold of the constrained peptide (Figure 5E). In addition, MP-594 maintained the same hydrogen bond formed as that which exists between the Trp of M06 and the carbonyl backbone group found on Leu^54^ of Mdm2. However, there were changes in the global conformation of MP-594 in relation to M06, whereby it translated further across the Mdm2 binding groove, resulting in a major change in the position of the Cα carbon backbone (Figure 5C,E). A similar displacement was observed in relation to the SAH-8 peptide (Figure 5D). These changes enable the side chain interactions of the critical residues to be maintained especially in relation to the d-Trp^7^ of MP-594, where the re-orientation of the Cα allows the indole side chain to optimally pack with Mdm2. Interestingly, overlay of MP-594 with the pMI (N8A) linear peptide (PDB: 3LNZ) showed an even greater change in the bound conformation of the peptide, where a longitudinal translation down the pocket can be seen in combination with a movement across the binding groove on Mdm2 (Figure 5F). This result demonstrates the cumulative effects that hydrocarbon stapling of the linear peptide and introduction of the d-Trp^7^ have on the conformation of the bound peptide. In the complex, the interaction of the hydrocarbon staple with the surface of Mdm2 and the accommodation of d-Trp^7^ in the target binding pocket causes the dramatic re-orientation of MP-594 with respect to pMI.

### 3.6. Cell Homogenate Proteolytic Stability of d-Amino Acid Modified Stapled Versus Linear Peptides

**Figure 6:**
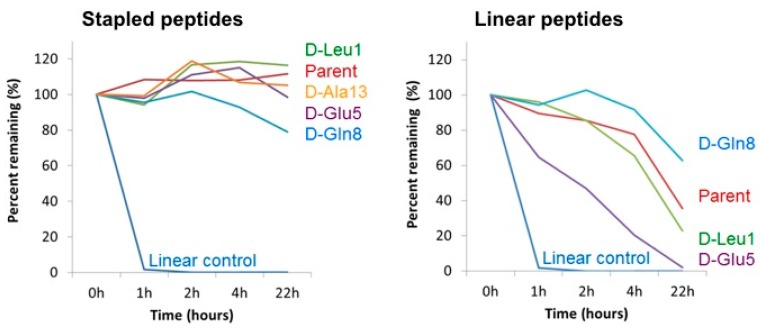
Stability of stapled and linear peptides in whole cell homogenate. Parent sequences correspond to MP-292 (left panel) and MP-189 (right panel). The linear control peptide is ONEG [38].

Amongst the known benefits of incorporating d-amino acids in peptides is their capacity to confer resistance to proteolytic degradation. To explore whether such a benefit was realized in the context of the D-amino acid modified peptides investigated in this study, we subjected a subset of both linear and stapled analogs of MP-292 to stability analysis in whole-cell homogenates (Figure 6). As expected, MP-292 showed minimal degradation over 22 h despite being exposed to a multitude of cellular proteases. Thus, in the context of MP-292, further proteolytic stabilization through d-amino acid substitution was not requisite, and similar stability profiles were found for those d-amino acid modified analogs studied. In sharp contrast, the linear peptides were all significantly less stable than their stapled counterparts. However, the Aib^4^ and Aib^11^ modified linear peptide MP-189 and d-amino acid modified analogs thereof displayed moderate stabilities (Figure 6). Interestingly, the d-Glu^5^ modified linear peptide analog showed decreased stability, perhaps a result of locally perturbed secondary structure. Indeed, replica exchange simulations suggested that d-Glu^5^ modification may compromise secondary structural stability, predicted as a result of H-bonding between the sidechains of Glu^5^ and Thr^2^.

### 3.7. Aib, N-Me-Amino Acid, Pro, and Gly Substitutions Had Minimal Effects on a-Helicity, Target Binding, and Cellular Activity

To further explore the effects of helix-inducing or helix-disrupting amino acid substitutions, a series of stapled peptide analogs of MP-082—an N-terminal Lys(N3)-modified ATSP-7041 analog—were evaluated (Table 2). This series included Aib, l-Pro, Gly, and N-methylated amino acid modification at varying positions. Although d-amino acid scanning studies showed a tolerance for α-helicity within the stapled peptide analog of MP-292, it was anticipated that Aib (helix-inducing) versus N-methylated amino acid, Pro, or Gly (helix-disrupting) modifications would exemplify significant differences in α-helicity, as determined by CD studies. However, such modifications did not result in a significant change in α-helicity relative to the parent sequence MP-081 (Table 2). Furthermore, several analogs in this series of peptides exemplified relatively potent Mdm2 binding affinities (i.e., *K_d_* < 100 nM), except for Aib^2^ (*K_d_* = 1,706 nM), N-Me-Thr^2^ (*K_d_* = 351 nM), N-Me-Ala^8^ (*K_d_* = 671 nM), Aib^8^ (*K_d_* = 119.4 nM) and N-Me-Gln^9^ (*K_d_* = 211 nM) substitutions. CD studies and MD simulations of this series of MP-081 analogs showed that they exhibited good α-helicity (e.g., >30% by CD). An explanation for the significantly decreased Mdm2 binding affinity of N-Me-Thr^2^ or Aib^2^-substituted MP-081 may be that it effected a local perturbation manifested in a loss of a critical H-bond between the backbone NH of Phe^3^ of MP-081 and the sidechain amide moiety of Mdm2 residue Gln72 (Figure S4). Substitutions that disrupt contiguous helix-stabilizing intramolecular H-bonds involving the backbone amides at positions 8 and 9 of MP-081 (i.e., Ala^8^ to N-Me-Ala^8^, Gln^9^ to N-Me-Gln^9^ or Pro^9^) decreased α-helicity to <30%, as measured by CD studies (Table 2). MD simulations predicted that N-methylation of Ala^8^ and Gln^9^ would disrupt α-helicity in both free and bound states (see further discussion with Figure S5). Both N-Me-Ala^8^ and N-Me-Gln^9^ substitutions abolished cellular activity (EC_50_ > 50 µM, Table 2). In contrast, Gln^9^ to Pro^9^ substitution with MP-081 maintained α-helicity (27.4%) and showed about three-fold lower Mdm2 binding affinity and six-fold decreased cellular potency (Table 2). Surprisingly, Gly substitutions at various positions were tolerated, with each of the MP-081 analogs tested showing α-helicities, Mdm2 binding affinities, and cellular activities similar to the parent sequence (a minor exception was the Glu^5^ to Gly^5^ substituted analog, which was determined to have 28.6% helicity). Noteworthy was the triple Gly-modified (i.e., Gly^1,5,8^-substituted) MP-081 analog (Table 2), which showed moderately reduced α-helicity (20.4%), retained high binding affinity to Mdm2 (Kd 49.8 nM,), and only three-fold lower cellular activity. Single Gly substitutions for Leu^1^, Glu^5^, Gln^9^, and Ser^12^ showed similar α-helicities and Mdm2 binding affinities relative to the parent sequence MP-081 (Table 2; see further molecular modeling discussion with Figure S6). Lastly, this series of Gly-substituted stapled peptides showed similar cellular potencies (ranging from 263.5–1697 nM), although the Gly^1^ analog and the triple Gly^1,5,8^ analog were approximately three-fold less potent than MP-081.

### 3.8. Membrane Permeability Correlates with Peptide Lipophilicity

**Figure 7:**
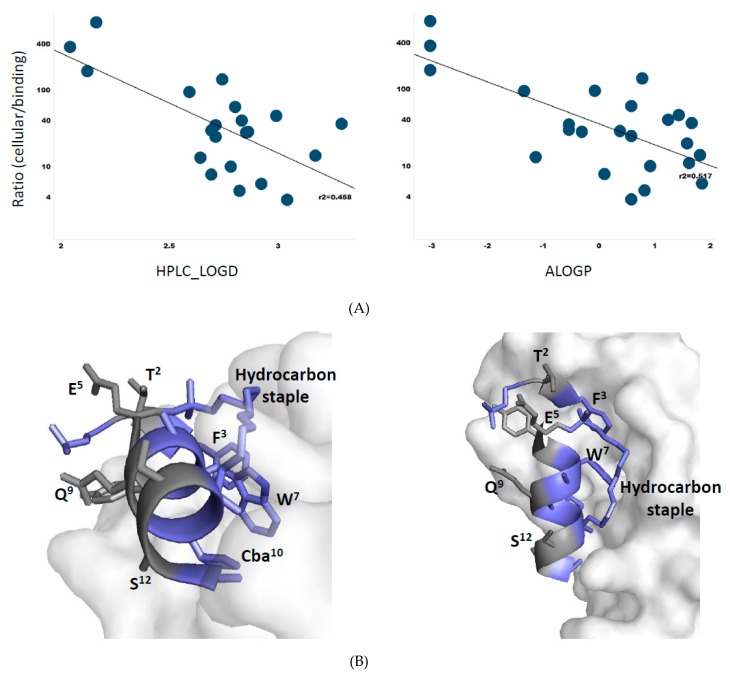
(**A**) Correlations between cellular/biochemical ratios (see Table 1 and Table 2) and lipophilicity determinations; HPLC-LogD (Left panel) and ALogP (Right panel). (**B**) α-Helical structure of ATSP-7041 showing it amphipathicity in terms of hydrophobic surface in blue (F^3^, W^7^, Cba^10^, hydrocarbon staple) that is interacting with Mdm2, whereas the hydrophilic surface in gray (T^2^, E^5^, Q^9^, S^12^) is exposed to the solvent. The X-ray crystal structure of ATSP-7041 complexed with Mdmx (PDB code, 4N5T) was used for this analysis.

Having amassed a sizable library of ATSP-7041 analogs (50 molecules), we looked for trends that might aid in the design of cellularly active stapled peptides. As an estimate for membrane permeability, we normalized peptide cellular potencies by their target-binding affinities (ratios listed in Table 1 and Table 2). A visual inspection of these values revealed several instances where substitutions that increased lipophilicity correlated with a decreased cellular/binding ratio compared to its corresponding parent peptide, MP-292 or MP-081 (e.g., E5A, Q8A, and S12 in Table 1; T2(Aib), E5G, Q9G in Table 2). To probe this relationship more formally, we plotted the cellular/biochemical ratios against both experimentally derived lipophilicity (HPLC-logD) and calculated lipophilicity (AlogP) and found a reasonable linear relationship (Figure 7A, R^2^ = 0.46 for HPLC-LogD and R^2^ = 0.52 for ALogP). Thus, lipophilicity, at least for this family of peptides, appears to be a primary driver of cellular uptake. It was noted that cell/target ratios in the range of 1000 to 1 are interpreted to represent low to high cellular permeabilities (assuming similar stabilities toward intracellular proteases and cell uptake mechanisms). It was further surmised that stapled peptides having greater lipophilicities (e.g., HPLC-LogD or ALogP values >3) were aligned with superior cell/target ratios (1–10 range). As shown in Figure 7B, the hydrophobic residues of ATSP-7041 are also engaged in target binding, whereas the hydrophilic residues are exposed to solvent. Therefore, the amphipathic properties of this series of stapled peptides translates in terms of both membrane partitioning and target binding to achieve cellular potency.

### 3.9. Conclusions: A Macrocyclic α-Helical Peptide Model System to Explore Biophysical and Cellular Permeability Properties

To establish ATSP-7041 analogs as a compelling model system to study stapled peptide biophysical properties, cellular activities, and membrane permeability, we focused on MP-292 as a prototypic example. Indeed, this molecule proved to be ideal as a library template, as it showed: (i) 1:1 stoichiometric binding to Mdm2; (ii) sub-µM potency in our cellular assay (p53 reporter assay); (iii) an absence of activity in our cellular counterscreen (p53-independent reporter assay); (iv) an absence of activity in a cell membrane disruption assay (LDH release); and (v) highly proteolytically stable in a cell homogenate assay. As exemplified above by both Ala-scanning and d-amino acid scanning analysis of MP-292, as well as Aib, N-methylated amino acid, L-Pro, and Gly substitutions of MP-081, there are compelling future opportunities to further explore the biophysical and cellular permeability properties of this macrocyclic α-helical peptide model system. We have shown that (within the framework of a stapled α-helical peptide) it is possible to incorporate d-amino acids as well as other classic helix-breakers, such as N-methylated amino acids, l-Pro, and Gly, to expand chemical space without abolishing α-helical conformational properties. Indeed, a surprising result uncovered here was the ability of MP-594 (the d-Trp^7^-modified stapled peptide analog) to maintain binding and cellular activity despite the change in stereochemistry at a key binding position—a result explained by modeling and a high-resolution X-ray crystal structure. Furthermore, the comparative analysis of the Aib^4,11^-substituted MP-292 analogs with respect to α-helicity, Mdm2 binding affinity, cellular activity, and proteolytic stability provide insight to understand the unique biophysical and cellular permeability properties of the benchmark stapled peptide ATSP-7041. Lastly, the lipophilic properties of this series of stapled peptides were investigated in terms of determining their experimental and computational partition coefficients (HPLC-LogD and ALogP, respectively). A good correlation between these partition coefficients and the cell/target ratios was observed. The importance of lipophilicity and permeability has been noted in other studies [32,33,34]. Together, these studies and ours suggest that simply increasing lipophilic character while maintaining strong a binding affinity may enhance peptide permeability and cellular activity. Since a prerequisite for cellular permeability is partitioning of the peptide into an apolar membrane, it is reasonable to assume that a more lipophilic peptide may achieve enhanced partitioning. However, surpassing what may be defined as a peptide-dependent threshold, LogD will likely result in undesired physicochemical properties, such as poor solubility, aggregation, and/or nonspecific binding. Special attention should be given to the judicious regiospecific placement of amino acids to achieve an optimal balance of hydrophobic and hydrophilic surface topology. Interestingly, upon examination of the MdmX:ATSP-7041 co-crystal structure, an amphipathic distribution of hydrophobic amino acids is seen. In particular, a hydrophobic face composed of the hydrocarbon staple moiety and target binding residues (Phe^3^, Trp^7^, and Cba^10^) is balanced by an opposing hydrophilic face (Thr^2^, Glu^5^, Gln^9^, and Ser^12^). We speculate that this spatial configuration may contribute to favorable aqueous-phase properties, membrane permeability, and ultimately to the sub-µM cellular activity determined for this molecule. Future work will focus more deeply on the cell uptake and intracellular exposure analysis, as well as further exploration of the relationship of lipophilicity to cellular permeability through the expanded chemical diversity of this macrocyclic α-helical peptide model system.

## Figures and Tables

**Table 1:** Sequence, helicity, cellular activity (EC50), and Mdm2 binding (Kd) of MP-292 and derivatives. Ratio refers to the fold-shift between the binding affinity and cellular activity. Section A) Alanine scan of MP-292 highlights F3, W7 and Cba10 as key residues for Mdm2 binding and cellular activity. Section B) D-amino acid scan of MP-292 shows that most variants maintain Mdm2 binding and cellular activity. Section C) D-amino acids substitutions to MP-189, a linear equivalent of stapled peptide MP-292, disrupt secondary structure and binding to Mdm2.

Table section	Peptide	N-term	1	2	3	4	5	6	7	8	9	10	11	C-term	Helicity (%)	Cellular (µM)	Mdm2 (nM)	Ratio
**A**	MP-292	Ac-	L	T	F	R8	E	Y	W	Q	L	Cba	S5	-SAA-amide	32.8	0.54	18.6	29
	L1A	Ac-	**A**	T	F	R8	E	Y	W	Q	L	Cba	S5	-SAA-amide	53.3	0.50	17.6	28
	T2A	Ac-	L	**A**	F	R8	E	Y	W	Q	L	Cba	S5	-SAA-amide	36.2	1.0	21.3	47
	F3A	Ac-	L	T	**A**	R8	E	Y	W	Q	L	Cba	S5	-SAA-amide	48.4	37	3600	10
	E5A	Ac-	L	T	F	R8	**A**	Y	W	Q	L	Cba	S5	-SAA-amide	27.6	0.18	29.8	6
	Y6A	Ac-	L	T	F	R8	E	**A**	W	Q	L	Cba	S5	-SAA-amide	50.3	0.55	38.9	14
	W7A	Ac-	L	T	F	R8	E	Y	**A**	Q	L	Cba	S5	-SAA-amide	ND	8.8	5093	2
	Q8A	Ac-	L	T	F	R8	E	Y	W	**A**	L	Cba	S5	-SAA-amide	33.4	0.10	26.5	4
	L9A	Ac-	L	T	F	R8	E	Y	W	Q	**A**	Cba	S5	-SAA-amide	48.7	0.16	12.0	13
	Cba10A	Ac-	L	T	F	R8	E	Y	W	Q	L	**A**	S5	-SAA-amide	ND	11	176	61
	S12A	Ac-	L	T	F	R8	E	Y	W	Q	L	Cba	S5	**-AAA-amide**	36.5	0.35	20.4	17
**B**	L1(D-Leu)	Ac-	**D-Leu**	T	F	R8	E	Y	W	Q	L	Cba	S5	-SAA-amide	44.1	0.6	31	18
	T2(D-Thr)	Ac-	L	**D-Thr**	F	R8	E	Y	W	Q	L	Cba	S5	-SAA-amide	40.7	3.4	136	25
	F3(D-Phe)	Ac-	L	T	**D-Phe**	R8	E	Y	W	Q	L	Cba	S5	-SAA-amide	51.8	>50	6485	>8
	E5(D-Glu)	Ac-	L	T	F	R8	**D-Glu**	Y	W	Q	L	Cba	S5	-SAA-amide	38.2	1.0	32	30
	Y6 (D-Tyr)	Ac-	L	T	F	R8	E	**D-Tyr**	W	Q	L	Cba	S5	-SAA-amide	43.2	3.2	286	11
	W6(D-Trp) (MP-384)	Ac-	L	T	F	R8	E	Y	**D-Trp**	Q	L	Cba	S5	-SAA-amide	41.0	0.6	30	20
	Q8(D-Gln)	Ac-	L	T	F	R8	E	Y	W	**D-Gln**	L	Cba	S5	-SAA-amide	27.1	2.1	22	95
	L9(D-Leu)	Ac-	L	T	F	R8	E	Y	W	Q	**D-Leu**	Cba	S5	-SAA-amide	39.0	1.1	30	37
	Cba10(D-Cba)	Ac-	L	T	F	R8	E	Y	W	Q	L	**D-Cba**	S5	-SAA-amide	ND	8.1	1650	5
	S12(D-Ser)	Ac-	L	T	F	R8	E	Y	W	Q	L	Cba	S5	**-(D-Ser)-AA-amide**	41.2	1.2	8.6	140
	A13(D-Ala)	Ac-	L	T	F	R8	E	Y	W	Q	L	Cba	S5	**-S-(D-Ala)-A-amide**	41.8	0.6	17	35
	A14(D-Ala)	Ac-	L	T	F	R8	E	Y	W	Q	L	Cba	S5	**-SA-(D-Ala)-amide**	34.6	0.5	18	28
**C**	Linear Parent (MP-189)	Ac-	L	T	F	Aib	E	Y	W	Q	L	Cba	Aib	-SAA-amide	35.3	33.5	43.1	777
	Linear L1(D-Leu)	Ac-	D-Leu	T	F	Aib	E	Y	W	Q	L	Cba	Aib	-SAA-amide	37.2	>50	277	>180
	Linear E5(D-Glu)	Ac-	L	T	F	Aib	D-Glu	Y	W	Q	L	Cba	Aib	-SAA-amide	21.4	>50	135	>370
	Linear Q8(D-Gln)	Ac-	L	T	F	Aib	E	Y	W	D-Gln	L	Cba	Aib	-SAA-amide	21.8	>50	119	>370
	Linear L9(D-Leu)	Ac-	L	T	F	Aib	E	Y	W	Q	D-Leu	Cba	Aib	-SAA-amide	19.7	>50	88	>568

**Table 2:** Sequence, helicity, cellular activity (EC50), and Mdm2 binding (Kd) of MP-081 and derivatives. A variety of putative helix inducers and helix-breakers are accommodated in the context of stapled peptide MP-081 with most variants maintaining helical structure, Mdm2 binding and cellular activity. Ratio refers to the fold-shift between the binding affinity and cellular activity.

Peptide	N-term	1	2	3	4	5	6	7	8	9	10	11	C-term	Helicity (%)	Cellular (µM)	Mdm2 (nM)	Ratio
MP-081	Ac-K(N3)-(βA)-	L	T	F	R8	E	Y	W	A	Q	Cba	S5	-SAA-amide	49.6	0.42	19.0	22
L1(Aib)	Ac-K(N3)-(βA)-	**Aib**	T	F	R8	E	Y	W	A	Q	Cba	S5	-SAA-amide	42.7	0.49	29.5	17
T2(Aib)	Ac-K(N3)-(βA)-	L	**Aib**	F	R8	E	Y	W	A	Q	Cba	S5	-SAA-amide	38.3	2.9	1706	2
A8(Aib)	Ac-K(N3)-(βA)-	L	T	F	R8	E	Y	W	**Aib**	Q	Cba	S5	-SAA-amide	37.5	1.5	119.4	12
Q9(Aib)	Ac-K(N3)-(βA)-	L	T	F	R8	E	Y	W	A	**Aib**	Cba	S5	-SAA-amide	28	0.25	33.1	8
S12(Aib)	Ac-K(N3)-(βA)-	L	T	F	R8	E	Y	W	A	Q	Cba	S5	**-(Aib)-AA-amide**	37.3	0.25	24.4	10
A13(Aib)	Ac-K(N3)-(βA)-	L	T	F	R8	E	Y	W	A	Q	Cba	S5	**-A-(Aib)-A-amide**	34.4	0.59	25.7	23
L1(N-methyl L-Leu)	Ac-K(N3)-(βA)-	**NMe-L**	T	F	R8	E	Y	W	A	Q	Cba	S5	-SAA-amide	32.6	0.46	23.5	20
L1(N-methyl L-Ala)	Ac-K(N3)-(βA)-	**NMe-A**	T	F	R8	E	Y	W	A	Q	Cba	S5	-SAA-amide	47.2	1.49	15.4	97
T2(N-methyl L-Thr)	Ac-K(N3)-(βA)-	L	**NMe-T**	F	R8	E	Y	W	A	Q	Cba	S5	-SAA-amide	38.2	7.7	352.2	22
A8(N-methyl L-Ala)	Ac-K(N3)-(βA)-	L	T	F	R8	E	Y	W	**NMe-A**	Q	Cba	S5	-SAA-amide	16.9	>50	671.3	>75
Q9(N-methyl L-Gln)	Ac-K(N3)-(βA)-	L	T	F	R8	E	Y	W	A	**NMe-Q**	Cba	S5	-SAA-amide	20.2	>50	211.2	>237
A13(N-methyl L-Ala)	Ac-K(N3)-(βA)-	L	T	F	R8	E	Y	W	A	Q	Cba	S5	**-S-(Nme-A)-A-amide**	38.6	1.6	10.0	158
A14(N-methyl L-Ala)	Ac-K(N3)-(βA)-	L	T	F	R8	E	Y	W	A	Q	Cba	S5	**-SA-(Nme-A)-amide**	47.5	0.71	5.9	120
L1P	Ac-K(N3)-(βA)-	**P**	T	F	R8	E	Y	W	A	Q	Cba	S5	-SAA-amide	41.6	0.75	21.9	34
Q9P	Ac-K(N3)-(βA)-	L	T	F	R8	E	Y	W	A	**P**	Cba	S5	-SAA-amide	27.4	3.1	77.1	41
S12P	Ac-K(N3)-(βA)-	L	T	F	R8	E	Y	W	A	Q	Cba	S5	**-PAA-amide**	30.8	3.7	45.7	80
L1G	Ac-K(N3)-(βA)-	**G**	T	F	R8	E	Y	W	A	Q	Cba	S5	-SAA-amide	44.6	1.6	22.1	73
E5G	Ac-K(N3)-(βA)-	L	T	F	R8	**G**	Y	W	A	Q	Cba	S5	-SAA-amide	28.6	0.26	61.8	4
Q9G	Ac-K(N3)-(βA)-	L	T	F	R8	E	Y	W	A	**G**	Cba	S5	-SAA-amide	38.8	0.68	42.0	16
S12G	Ac-K(N3)-(βA)-	L	T	F	R8	E	Y	W	A	Q	Cba	S5	**-GAA-amide**	46.5	0.65	18.9	34
L1G, E5G, A8G	Ac-K(N3)-(βA)-	**G**	T	F	R8	**G**	Y	W	**G**	Q	Cba	S5	-SAA-amide	20.4	1.7	49.8	34

## Supplementary Materials

The Supplementary Materials are available online.
