# Supplementary material for: Incorporation of Putative Helix-Breaking Amino Acids in the Design of Novel Stapled Peptides: Exploring Biophysical and Cellular Permeability Properties
Source: Molecules. 2019 Jun 20;24(12):2292. doi: 10.3390/molecules24122292 (PMC6632053; doi:10.3390/molecules24122292)
Supplement: Supplementary file 1 [file molecules-24-02292-s001.zip › molecules-521495-supplementary-FINAL/molecules-521495-supplementary-final.pdf]

Supplementary Section

Table S1: Data collection statistics and refinement parameters.

Property	6AAW
Resolution (Å)	40.33 - 2.00 (2.11 – 2.00)
Space Group	C 12_12
Unit Cell Dimensions (Å)	a= 80.95, b= 43.38 Å, c= 34.54 Å (a= 90° b=94.82°, g=90°)
Temp (K)	100
Redundancy	6.3
Unique Collected Reflections	8132
Completeness (%)	99.3 (95.2)
R Pim (%)	11.0 (53.0)
I/sigma	5.7 (3.4)
R factor (%)	18.41
R free (%)	22.33
RMS Bonds (Å)	0.01
RMS Angles (°)	2.2
Wilson B-factor ($Å^2$)	18.3
Average Refined B Factors	
Chain A (Mdm2)	26.75
Chain B (Peptide)	22.55
Waters	35.34
Number of Water Molecules	40
Ramachandran Data (Rampage). Number of Residues in(%) :	
Favoured Region	98.8
Allowed Region	1.2
Outlier Region	0

S1: Modeling the binding of MP-292 with Mdm2: We generated a model of the stapled peptide ATSP-7041 bound to Mdm2 based on the available crystal structures of ATSP-7041 bound to MdmX (PDB id 4N5T). Peptide residues A^8, Q^9 were mutated to Q8, L9 and the models were subjected to MD simulations for further refinement. The bound conformation of the MP-292 peptide and Mdm2 protein remained stable with rmsd < 2Ang against the starting conformation. The peptide engages in several hydrogen bonds with the protein, as seen in the original p53-Mdm2 structure (Figure S1) including between 1) the side chain N of Trp^7 and the carbonyl backbone of Leu^{54}; 2) the backbone N of Phe^3 and sidechain O of Gln^{72} and are preserved in the simulations. In addition, there are several transient H-bond interactions involving Lys^{52}, Arg^{98}, Tyr^{101} of Mdm2 and the backbone atoms of Ser^{12}, Ala^{13}, Ala^{14}. In addition, the peptide is also stabilized by hydrophobic interactions mediated by Phe^3, Trp^7, and Cba^{10} Next, we investigated the conformational landscape of the free MP-292 peptide in solution. Biasing Potential Replica Exchange MD [ ] simulations showed that the free peptide exhibited increased flexibility with RMSD ranging between 2 to 6 Å sampling several partially unfolded conformation. Secondary structure calculations revealed that only ~40% of the peptide is α-helical in solution, which is

in good agreement with the CD data. The regions within the stapled linker are relatively more helical and the increased disorder arises from these flanking regions.

Table S2. Binding affinity measured by surface plasmon resonance
[a] Dissociation constant is obtained by kinetically fitting the sensorgram with 1:1 binding model;
[b] Dissociation constant is obtained from the steady-state analysis; [c] not applicable due to steady-state analysis.

Peptide	K_D (nM)[a]	k_a ($M^{-1} s^{-1}$)	k_d (s^{-1})
MP-292	1.4	2.6×10^5	3.6×10^{-4}
(F3A)	1800[b]	NA[c]	NA[c]
(W7A)	5000[b]	NA[c]	NA[c]
(Cba10A)	50	3.3×10^5	0.016
(D-Phe3)	>5000[b]	NA[c]	NA[c]
(D-Trp7)	5	1.8×10^5	9.1×10^{-4}
(D-Cba10)	340	1.2×10^5	0.04
MP-081	1.9	1.5×10^5	3.0×10^{-4}

S2: Exploration of the specific mechanisms for affinity loss in F10 to D-Phe and Cba10 to D-Cba by MD simulations: Introduction of D-Phe3 resulted in unfolding at the N-terminus of the peptide (bound-state alpha helicity decreased from ~99% to 55%, Figure S2) and a critical hydrogen bond between the backbone amide of Phe^3 and the sidechain carbonyl of Gln^{68} was no longer observed in the bound state, resulting in reduced affinity. Upon the introduction of D-Cba^{10}, the altered stereochemistry of the sidechain would cause steric clashes with the sidechain of His^{97} of Mdm^2; the peptide adapts to this for binding by losing alpha helicity at its C- terminus, especially within the stapled region; this results in the observed loss of affinity.

Supplementary Figure Legends:

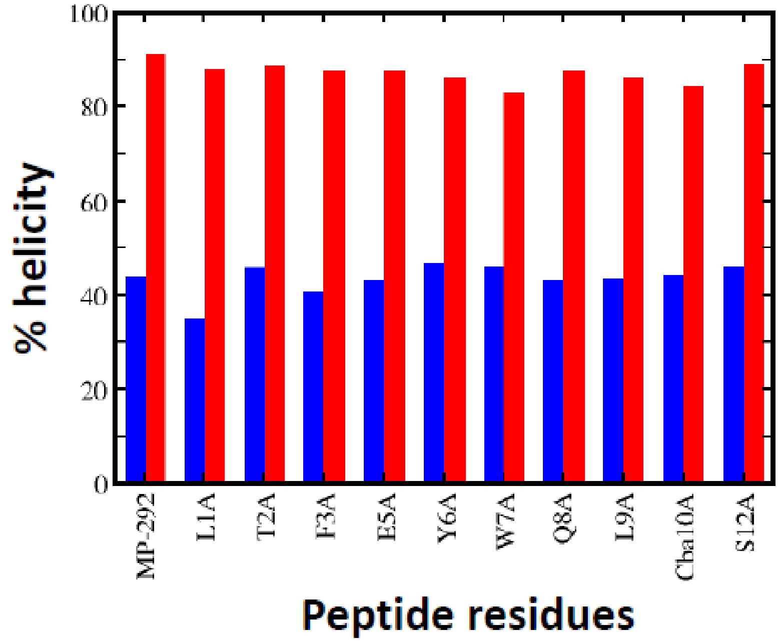

Figure S1: Helicity of apo versus bound peptide. Peptide helicity is calculated using the DSSP program using the conformations sampled during HREMD (apo) and MD (complex) simulations.

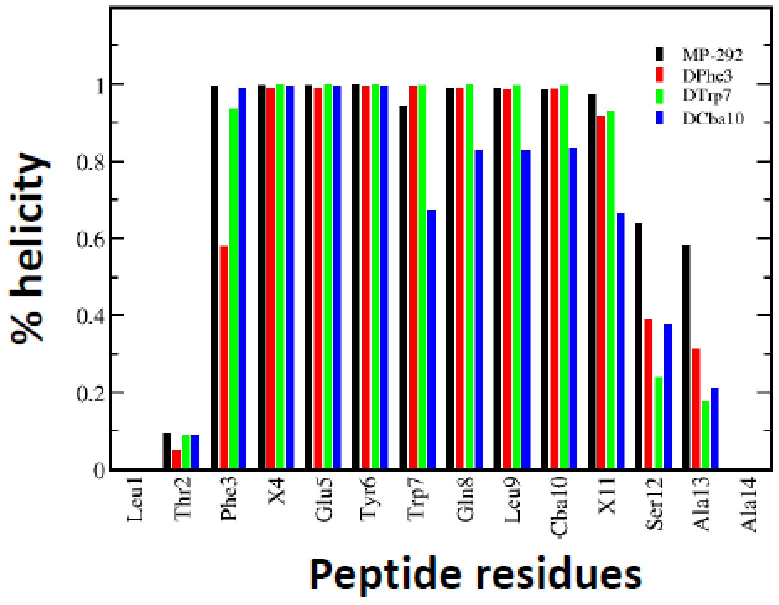

Figure S2: Residue-wise α-helicity of MP-292 and D-amino acid substituted MP-292 peptides.

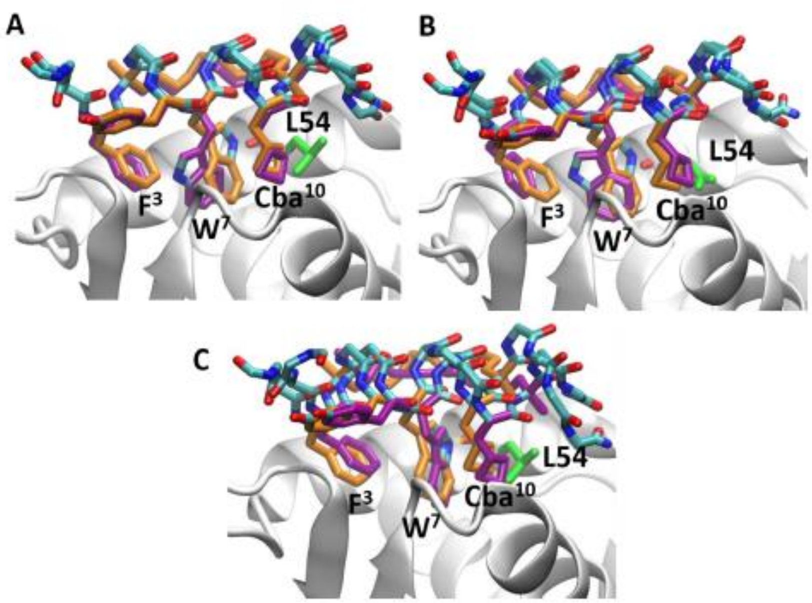

Figure S3: MD snapshot showing the binding of MP-594 with Mdm2. Superposition of the WT stapled peptide (Orange carbon) with D-Trp7 substituted (violet carbon) (A) starting structure of D-Trp7 substituted peptide for the MD simulation (B) intermediate structure (C) most populated structure of D-Trp7 substituted peptide sampled during MD simulations. The rest of the peptide residues are colored in cyan and the bound Mdm2 is shown as cartoon (Grey) with the Leu54 is highlighted in green sticks.

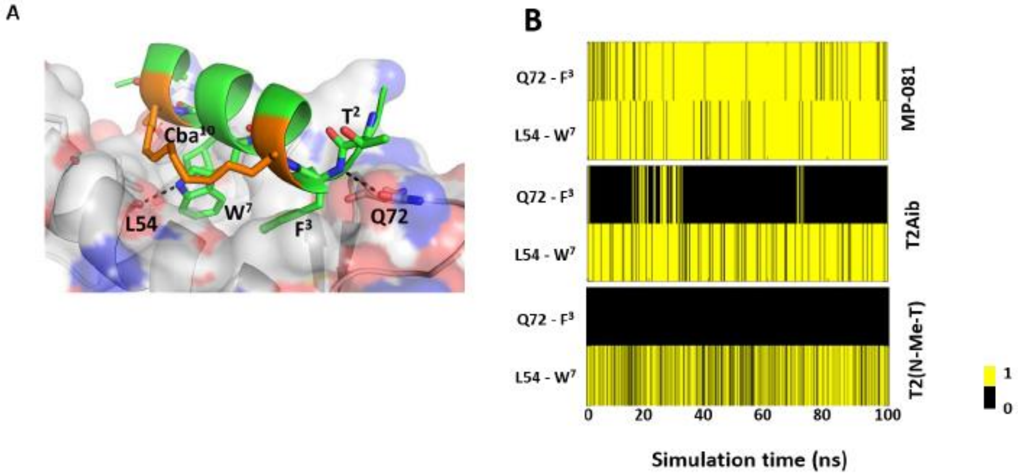

Figure S4: (A) Model of MP-292 in complex with Mdm2. Mdm2 is shown as surface (Grey color) and the bound peptide (Green carbon) is shown as a cartoon. Peptide residues, staple linker (orange color) and interacting residues from Mdm2 are shown as lines with Mdm2/MP-292 hydrogen bond interactions are shown as dashed lines (black). (B) Time evolution of Protein – peptide h-bond interactions from conformations sampled during MD (complex) simulations with color yellow and black corresponds to the presence and absence of H-bonds.

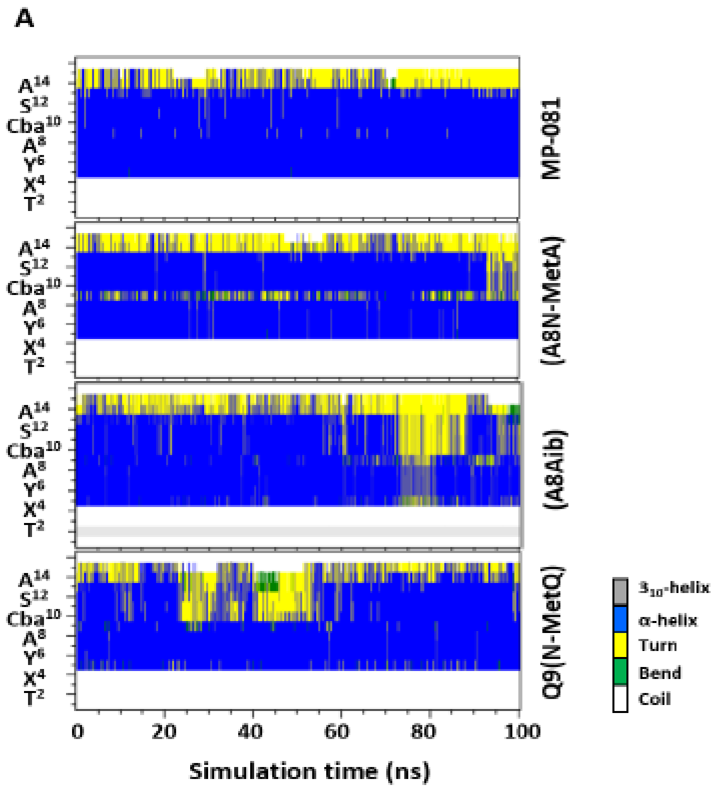

Figure S5: Evolution of the secondary structure of MP-081 analogs calculated using the DSSP program for conformations sampled during the MD simulations in the presence of Mdm2. Secondary structure (blue for α-helix, grey for 310-helix, yellow for turn, green for bend, and white for coil) along the protein chain (y-axis) vs simulation time (x-axis).

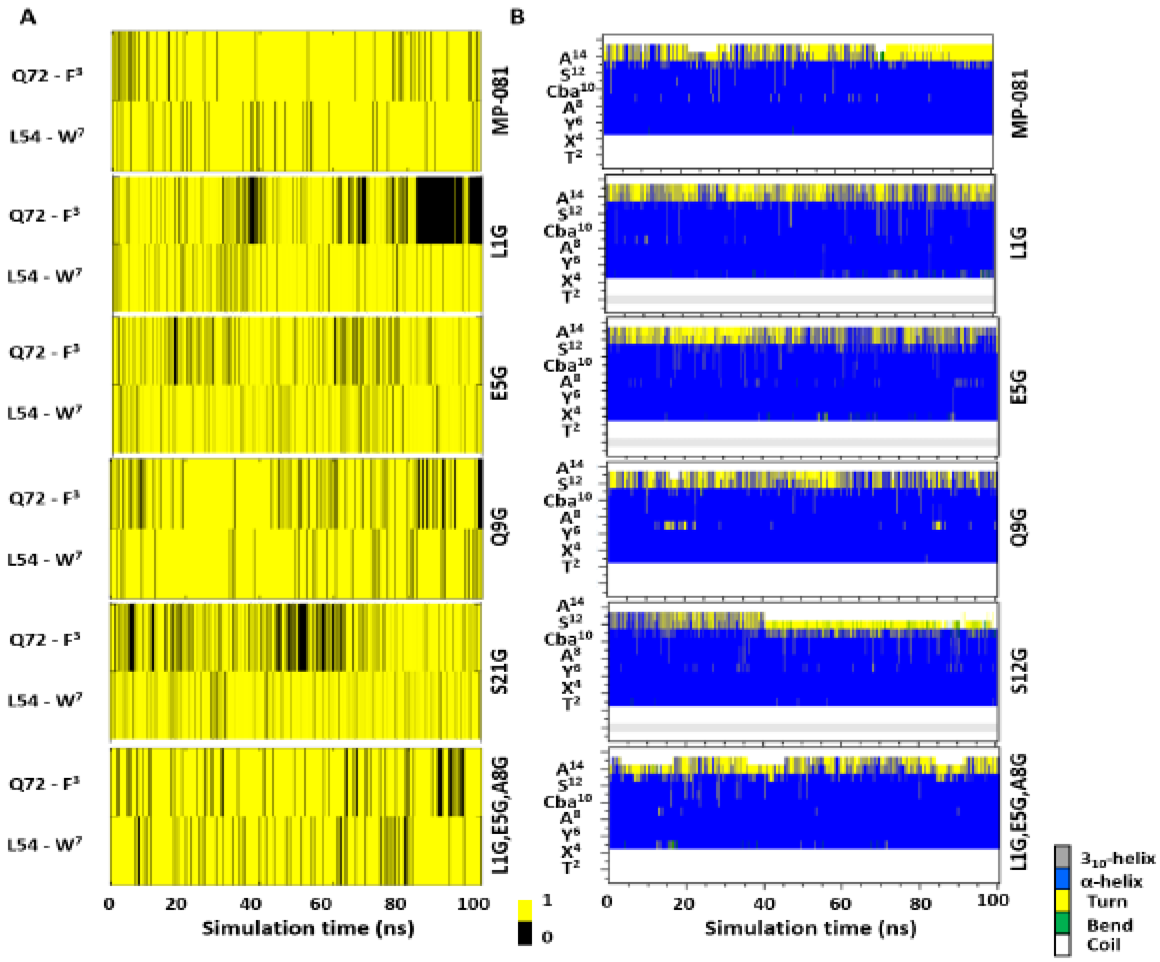

Figure S6: (A) Time evolution of protein–peptide H-bond interactions from conformations sampled during MD (complex) simulations with yellow and black colors corresponding to the presence or absence of H-bonds, respectively. (B) Evolution of the secondary structure of MP-081 analogs calculated using DSSP program for conformations sampled during the MD simulations in the presence of Mdm2. Secondary structure (blue for α-helix, grey for 3-10-helix, yellow for turn, green for bend, and white for coil) along the protein chain (y-axis) vs simulation time (x-axis).

Movie1: MD simulations of MP-594 (peptide shown in liquorice) bound to Mdm2 (not shown for clarity) showing the conformational change of the sidechain in D-Trp^7 together with the translation of the backbone as the Trp^7 sidechain reorients to a conformation similar to that adopted by L-Trp^7. The sidechain torsion angles (χ1, χ2) of the D-Trp^7 sidechain change from the initial model (81°, -101°) to final (156°, 18°); in contrast the torsion angles of the L-Trp^7 sidechain are (-161°, -86°). It is clear that the formation of the hydrogen bond between the NH of the Trp^7 sidechain and the backbone carbonyl of Leu^{54} in both L and D forms coupled with χ1 values that are the same in magnitude but opposite in sign necessitates the differences between the χ2 values. The peptide with orange carbons is the reference peptide while the peptide with the magenta carbons is the D-Trp^7 containing peptide.

Movie2: MD simulations of the MP-594 (peptide shown in liquorice) bound to the N-terminal domain of Mdm2 (shown in cartoon) showing the conformational change of the sidechain in D-Trp^7 together with the translation of the backbone as the Trp7 sidechain reorients to a conformation similar to that adopted by L-Trp^7. The sidechain torsion angles (χ1, χ 2) of the D-Trp^7 sidechain change from the initial model (81°,

-101°) to final (156°, 18°); in contrast the torsion angles of the L-Trp^{7} sidechain are (-161°, -86°). It is clear that the formation of the hydrogen bond between NH of the Trp^{7} sidechain and the backbone carbonyl of Leu^{54} in both L and D forms coupled with $\chi 1$ values that are the same in magnitude but opposite in sign necessitates the differences between the $\chi 2$ values. The peptide with orange carbons is the reference peptide while the peptide with the magenta carbons is the D-Trp^{7} containing peptide.
